# Volunteering on Heritage at Risk sites and wellbeing: A qualitative interview study

**DOI:** 10.1111/hex.13852

**Published:** 2023-08-17

**Authors:** Julie Pattinson, Despina Laparidou, Joseph Akanuwe, Anna Scott, Claudia Sima, Carenza Lewis, Niro Siriwardena

**Affiliations:** ^1^ Community and Health Research Unit (CaHRU), School of Health and Social Care, College of Social Science University of Lincoln Lincoln UK; ^2^ Primary Care and Public Health Research, Community and Health Research Unit (CaHRU), School of Health and Social Care, College of Social Science University of Lincoln Lincoln UK; ^3^ University of Lincoln College of Arts and Programme Manager Centre for Culture and Creativity University of Lincoln Lincoln UK; ^4^ Department of Marketing Languages and Tourism, Lincoln International Business School University of Lincoln Lincoln UK; ^5^ University of Lincoln Lincoln UK; ^6^ Director of the University of Lincoln Community and Health Research Unit (CaHRU), School of Health and Social Care College of Social Science Lincoln UK

**Keywords:** heritage, motivations, physical, psychological, risk, volunteering, wellbeing

## Abstract

**Introduction:**

We explored experiences of volunteering in Heritage at Risk (HAR) projects, intended to mitigate the deterioration to historic assets, and the relationship with wellbeing. We aimed to understand the value of HAR to volunteers' wellbeing and relationships between HAR programme characteristics such as location, asset type and type of activity.

**Methods:**

We used a qualitative design with semi‐structured interviews of a purposive sample of volunteers recruited via Historic England (HE), employing Systematic Grounded Theory involving open, axial and selective coding.

**Findings:**

We interviewed 35 volunteers (18 male and 17 female) participating in 10 HAR projects. We identified six themes from the data analysis. (1) *Purpose*—was associated with volunteering motivations; there were some barriers to volunteering and many types of facilitators, including accessibility to local heritage sites. (2) *Being*—volunteers showed an appreciation and attachment to their place of residence. (3) *Capacity*—to learn heritage‐specific skills and diversify experiences in learning new skills (life, technical and personal). (4) Sharing—community engagement, connectedness, and inclusivity captured diversity and inclusion within volunteers across age, ethnicity, ability, and gender. (5) *Self‐nurture*—HAR volunteering created physical, psychological, and social benefits with limited risks and adverse outcomes. (6) *Self‐actualisation*—described volunteers reflecting on their experiences.

**Conclusion:**

HAR volunteering was associated with positive physical, social and psychological wellbeing outcomes. The study provides an evidence base for specific wellbeing benefits of volunteering at Heritage at Risk sites, although we could not conclude that HAR project activity was the cause of increased wellbeing.

**Public Contribution:**

Staff from HE were involved in designing the project brief. In selecting the HAR project sites, we took advice and recommendations from HE staff across all their six regional offices.

## 1 INTRODUCTION

Previous studies have indicated that volunteering on heritage sites has the potential to improve quality of life by offering perceived social and economic benefits and widening social cohesion.^1^ Historic England's 2022 strategy has advanced these understandings reviewing the evidence to explore wider theoretical insights into the wellbeing associations of heritage volunteering from a public health perspective.^2^

Heritage tells the story of who we are, connects us to others, provides us with a shared identity and collective memories, and attaches us to place.^1^ Heritage conservation involves ‘preserving’ an asset from harm and creating cultural ‘products’ to conserve heritage, including voice recordings of oral histories, poster exhibitions, heritage trails maps, books and murals.^3^ ‘Heritage at Risk’ (HAR) is a term given by Historic England to heritage assets identified as vulnerable to deterioration.^2^

Some HAR assets have benefitted from interventions to arrest or reverse deterioration, and some of these ‘HAR interventions’ have provided opportunities for volunteering by members of the public.^2^ The heritage sector has a tradition of working with and supporting volunteers, reporting that 5.5% of adult volunteers in England had undertaken heritage‐related activities, from formal roles in heritage trusts and boards, to informal support promoting and sharing local history and heritage.^1^

Heritage sites may positively impact on wellbeing as historic buildings, ancient monuments and traditional practices are associated with events, stories, communities, political movements that may affect wellbeing through emotional benefits, including increased attachment to place, feelings of security or comfort gained from a long‐term perspective and enhanced attachment to others through shared personal stories.^4^ Visiting heritage sites has been shown to ease challenges associated with dementia^1^ and the COVID‐19 pandemic.^5^

Since 2019 the National Heritage Lottery Fund in the United Kingdom has specified ‘People will have greater wellbeing’ as one of their required outcomes for funded heritage projects.^6^ Historic England also now aims to ensure everyone can experience the wellbeing benefits of heritage. They produced an assessment that evidenced the role of the historic environment in promoting health and wellbeing and looked at the ways in which that relationship could be explored further.^7^

Wellbeing is defined as quality of life and prosperity, positive physical and mental health, and sustainable, thriving communities.^8^ Wellbeing is also about ‘feeling good and functioning well’, as recognised as a vital part of health in the founding principles of the World Health Organization in 1948 stated that ‘Health is a state of complete physical, mental and social well‐being and not merely the absence of disease or infirmity’.^9^

In 2020 Heritage and Society,^1^ reporting on the value of heritage to society, individuals and community groups across England, discussed how the COVID‐19 pandemic had impacted on heritage volunteering. The majority of volunteers affected were in at‐risk groups for COVID‐19 infection, halting activities, and potentially putting five million people at additional mental health risk.^10^

It remains unclear whether heritage is associated with wellbeing in ways which other volunteering is not. Current research has shown limited evidence linking heritage volunteering directly with wellbeing. For instance, in 2010 a large‐scale review of volunteers on 134 Heritage Lottery Funded (HLF) projects which included visits to 27 projects across the United Kingdom, in‐person interviews with 224 volunteers and 725 responses to a survey, demonstrated a positive correlation between heritage volunteering and greater wellbeing ‘far higher’ compared to the nonheritage volunteering population. It reported younger HLF volunteers increased their skills and development and HLF unemployed volunteers were more likely to have participated in further training or education following their volunteering experience. However, it found limited evidence that the social outcomes could be attributed specifically to the heritage character of the projects.^11^

Employing Grounded Theory and an inductive approach helped to understand the relationship between different types of HAR volunteering, and their effect on wellbeing.^12^ Systematic Grounded Theory enabled us to consider existing knowledge and that HAR volunteering may be associated with positive physical, social, and psychological wellbeing outcomes, including differing motivations.^13^, ^14^

Table 1 shows the context of HAR sites and Table 2 shows the different types of heritage assets we included, such as outdoor activities on sites and monuments and indoor activities in museums and civic amenities. It is noted that this study was published from a wider study.^15^

**Table 1:** Context of HARAW project sites.

Project name and location	Context of HARAW project intervention
The Physic Well, Barnet, London	Remedial HAR work to the building involving a partnership of voluntary organisations, the borough, and Historic England. Monument came off the HAR register in 2019. Leased to Barnet Museum Charitable Trust and run by their volunteers.
Garrison Church of St George (1863), Royal Artillery Barracks, Woolwich, London	A collaboration between Historic England, the Heritage of London Trust, London Historic Buildings Trust, and the Woolwich Garrison Church Trust; WGCT to repair damage and make the building weather resilient. Site is now run by volunteers from the WGCT.
The Monumental Improvement Project, Cornwall	A collaboration between Historic England, the AONB and local community heritage groups clearing and conserving a variety of historic monuments in Cornwall. Strong partnership working has developed ongoing projects.
Adopt a Monument Scheme, Dartmoor	Project aimed to train volunteers in skills for heritage conservation of sites and monuments in Dartmoor National Park including 15 at‐risk monuments, with chances to gain certificated skills. Has built capacity for other conservation projects.
Australia Map, Wiltshire	Small‐scale, community‐initiated project, clearing vegetation from a WW1 chalk‐cut hillside monument, recutting features and replacing chalk on an unusual type of heritage asset.
Tilty Abbey, Essex	Project consolidating the last surviving walls of the abbey and improving its presentation led to the founding of the lively and ongoing Tilty Abbey Local History Group.
Mosely Road Baths, Birmingham	A renowned ‘Arts and Crafts’ civic building was repaired enabling it to remain open for public swimming. Now run by community volunteers and hosting fundraising activities.
North York Moors Monument Management Scheme, Yorkshire	Condition monitoring, conservation, and remedial work of archaeological sites. Large numbers of volunteers achieved significant reduction in the numbers of at‐risk and improvements to many others.
Allen Smelt Mill, Northumberland	Project removing damaging vegetation and consolidating walls carried out by self‐organised volunteers working with site manager and specialist contractors to take project beyond its original scope.
Anfield Cemetery, Liverpool	Friends of Anfield Cemetery maintain and represent the cemetery and ran ‘Lifting the Lids’ researching and installing interpretation boards, to present the site history and help young people learn various skills.

Abbreviations: HAR, Heritage at Risk; HARAW, Heritage at Risk and Wellbeing; WGCT, Woolwich Garrison Church Trust.

**Table 2:** HARAW: Heritage at Risk and Wellbeing Project characteristics.

HAR Project location	HAR asset	HAR asset in rural or urban setting	HAR asset is largely intact or in ruins	HAR volunteer activity indoors or outdoors	HAR volunteers improved physical condition of asset	HAR volunteers improved public access to or their appreciation of asset	HAR volunteer activity physically demanding	HAR volunteer activity directed by others
Physic Well, Barnet, London (PWBL)	17th century wellhouse	Urban	Intact	Indoors	No	Yes	No	No
Garrison Church Royal Artillery Barracks, Woolwich (GCRABWL)	19th century church	Urban	Ruins	Outdoors	No	Yes	No	No
Monumental Improvement Project, Cornwall (MIPC)	Multiperiod, 40 sites	Rural	Ruins	Outdoors	Yes	Yes	Yes	Yes
Adopt a Monument Scheme Dartmoor (AMSD)	Prehistorian burial mounds	Rural	Ruins	Outdoors	Yes	Yes	Yes	Yes
Luton Hoo Walled Garden, Bedfordshire (LHWGB)	18th century walled garden	Rural	Intact	Outdoors	No	No	Yes	Yes
Australia Map, Wiltshire (AMW)	World War One chalk‐cut hill figure	Rural	Intact	Outdoors	Yes	Yes	Yes	No
Tilty Abbey, Essex (TA)	Medieval Cistercian Abbey	Rural	Ruins	Outdoors	Yes	Yes	No	No
Mosely Road Swimming Baths, Birmingham (MRSBB)	Late 19th century Civic Building	Urban	Intact	Indoors	No	Yes	No	No
North York Moors Monument Management Scheme Yorkshire (NYMMMS)	Prehistoric burial mounds	Rural	Ruins	Outdoors	Yes	Yes	Yes	Yes
Allen Smelt Mill, Northumberland (ASM)	17th/18th century lead smelt mill	Rural	Ruins	Outdoors	No	Yes	Yes	No
Birkrigg Common Stone Circle, Cumbria (BCSS)	Neolithic/Bronze Age, Stone Circle	Rural	Ruins	Outdoors	Yes	Yes	Yes	Yes
Anfield Cemetery, Liverpool (AC)	19th/20th century civic cemetery	Urban	Intact	Outdoors	No	Yes	No	No

Abbreviation: HAR, Heritage at Risk.

## 2 METHODS

### 2.1 Design

We used a qualitative design employing Systematic Grounded Theory which uses a set of systematic inductive methods aimed towards theory development, involving the three steps of open, axial and selective coding.

### 2.2 Setting and participants

In 2020 we recruited a purposive sample via Historic England volunteer programme team leaders at 12 HAR community volunteer groups across England (Table 1).

### 2.3 Characteristics of the project sites

We used a current logic model which linked wellbeing to the historic environment and included how different routes into historical volunteering related to wellbeing outcomes.^16^ We chose the 10 HAR project sites (Table 2) because the site characteristics were related to the logic model wellbeing outcomes.

On receipt of expressions of interest in participating in the study, individuals were contacted by phone or email. They were briefed verbally and provided written information on what would be expected of them, their responsibility during the study, and their ethical rights. After the procedure of informed consent in which the participant read, were asked if they understood and then signed the informed consent document, the interview began. Participants were reminded that they were able to withdraw from the study at any time and remove their data after participation but before analysis.

Before the interview, we asked participants to answer questions on their demographic characteristics. Interviews lasted between 20 and 40 min. The semi‐structured interview schedule consisted of open‐ended questions and subsequent questions being developed in line with the information from participants' responses, until *theoretical saturation* was achieved. In Systematic Grounded Theory, data analysis has a well‐defined process that begins with basic description and moves to conceptual ordering and then on to theorising.^14^

The interview questions were designed to explore how participants felt about where they lived and their local heritage. We also explored the impact on wellbeing and HAR volunteering including impact across local communities.

All participants were assured of anonymity and confidentiality. After the interview participants were fully debriefed. Questions about demographic characteristics, including employment status, length of involvement in the current HAR project and ethnicity were asked at the beginning of the interview. Participants were provided with an identification number during their debriefing. Interviews were conducted online or over the phone and were recorded with a digital audiotape recorder. Trint software was used for transcribing interviews to create text documents, and there were validated for accuracy by the researchers (J. P., D. L., C. S., J. A.).

### 2.4 Ethical approval

The study was given ethical approval by the University of Lincoln Research Ethics Committee and all research was undertaken in accordance with the University of Lincoln code of practice for Research.

### 2.5 Analysis

We adopted a Straussian approach for data analysis^14^ and for theory to emerge, creating an understanding about HAR volunteering and wellbeing. Data were analysed and supported by NVivo 12 software.

Transcripts were systematically analysed putting aside any presuppositions and previous knowledge. Analysis began with open coding line by line to allow grounded codes to emerge from the data.^14^ The objective of open coding was to identify patterns in the data and to generate a multitude of categories to aid the identification of important concepts requiring further investigation. The categories were sometimes words elicited by the participants themselves, referred to by Strauss and Corbin^14^ as ‘in vivo’ language. The conceptual patterns that emerged from open coding guided the researchers to where to focus further analysis.

The second stage of data analysis was axial coding, achieved through systematic analysis and constant comparison of data. In axial coding, four analytical processes occurred: (1) continually relating subcategories to a category, (2) comparing categories with the collected data, (3) expanding the density of the categories by detailing their properties and dimensions, and (4) exploring variations in phenomena. Strauss and Corbin^14^ ^(p.123)^ described axial coding as ‘the process of relating categories to their subcategories, linking a category at the level of properties and dimensions’. A coding paradigm involving conditions, actions and interactions, and consequences actualises this process. The focus of axial coding in this study was to create a model that detailed the specific conditions that gave rise to a phenomenon's occurrence.^14^ The final stage of the three‐stage coding process was selective coding.

We analysed the data until theoretical data saturation was achieved. Glaser and Strauss^12^ when developing Grounded Theory defined data saturation as the point where ‘no additional data are being found whereby the [researcher] can develop properties of the category’. Finally, any field notes or memo cards, of the first author were evaluated by the additional authors to observe whether they were representative of the data transcripts.

## 3 RESULTS

We interviewed 35 HAR volunteers (18 male and 17 female) from 12 different HAR projects (Table 2 shows HAR project characteristics). Of the sample 24 were married, and most (24/35) were White British. Ages ranged from 20 to 80 years with an average age of 59.7 years. Most participants (32/35) were retired or reported being in full‐time employment. Participants' length of involvement in their current HAR project ranged from 2 months to 13 years. One participant self‐identified as disabled. We defined the definition of an older person as 65 years or above.^17^

**Figure 1:**
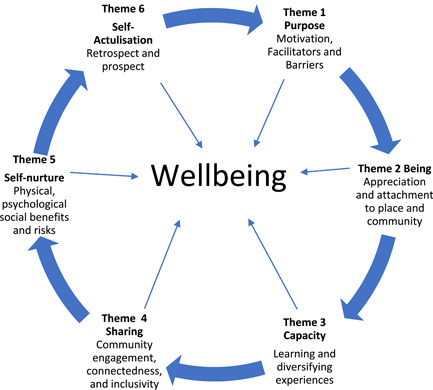
Six emergent themes identifying different types of wellbeing benefits of HAR volunteering. HAR, Heritage at Risk.

Six core themes emerged and were identified from the data shown in Table 3 and Figure 1.

**Table 3:** Emergent themes and subcategories from selective coding: core motivations and experiences of HAR volunteers.

*Theme 1: Purpose; Motivation, barriers, and facilitators to volunteering*
Motivation
To occupy time
Desire to preserve and restore historic sites
Local, social, family, education connections or placements
Involving and giving back to the community
Helping the community in a place they love
Connect with the natural environment
Contributing Skills
Expand skills or experiences
Barriers
Deficient resources, staff, funding, management, and bureaucracy
Differences across age groups
Too busy to do more
Time commitment (work, family) & seasonality
Physical health constraints
Other priorities—domestic and financial responsibilities
Natural or man‐made environmental barriers (physical impediments)
Facilitators
Funding and resources
Flexible scheduling and timetables
*Theme 2: Being; Appreciation and attachment to place*
Public's attachment to places
Dislikes and alienation about place
Enjoyment and satisfaction
Connection with history heritage and site
Volunteering as self‐expression (range and variety)
*Theme 3: Capacity; Learning and diversifying experience*
Enjoyment of learning about history and site through experience and from others
Experiencing different activities
Gaining new skills. knowledge, experiences, and qualifications
Communications skills
Technical skills
Undertaking different heritage roles
Has not developed new skills
*Theme 4: Sharing; Community engagement, connectedness, and inclusivity*
Community engagement and connectedness
Community ownership and legacy
Developing or expanding tourism
Approaches to engaging communities
Diversity and Inclusion
Age inclusivity
Cultural inclusivity
Ability inclusivity
Intersectional inclusivity
Gender inclusivity
Dissemination recruitment and spreading the word
Lack of public awareness
Promoting site and heritage to local community
*Theme 5: Self‐nurture; Physical, psychological, and social benefits: Risks, negatives & adverse outcomes*
Minor adverse effects on wellbeing
Psychological stress of volunteering or not feeling part of the community
No Negative effects or Minor quibbles
Benefits to wellbeing outweigh disadvantages
Promotes physical activity
Promotes psychological benefits
What you don't enjoy working in this project
Promotes social benefits
Increases social interaction—quantity of relationships
Socially rewarding—quality of the relationships
*Theme 6: Self‐actualisation; Retrospect and prospect*
Volunteering into the future
Strengthens working relationships
Volunteer's personal reflections
What do you feel you achieved being part of the project
Restored heritage is changing perceptions

Abbreviation: HAR, Heritage at Risk.

### 3.1 Theme 1—Purpose: Motivation, facilitators and barriers

#### 3.1.1 Motivation

Volunteers were motivated to occupy their time with a useful purpose and were likely to live nearby, they gave heritage their attention by protecting, preserving, and restoring historical sites.

It was started as I enjoy going out, walking, and then because I'm single, live alone. I did once I tired, I had time to use usefully. (HAR02)

And if it was in York, it was very convenient because it being for the community, you had to actually live nearby.

I think the building itself just required my attention. (HAR03)

Volunteers found participating in HAR projects convenient. They were motivated to ‘join in’ when family members were already volunteers. There were educational connections, including university student placements, while others ‘found out’ from local social events or word of mouth. Volunteers had a mixed skill set.

I knew of the existence of the association. My sister was already a member. (HAR04)

So, through the university, my course, actually, you have to do a placement somewhere. (HR05)

Volunteers wanted to give something back to the community and did this by collaborating and sharing their skills and were motivated as they felt they were doing something *noble* with *social value*.

I think it's also about sharing because no man is an island. So, it's not about just giving back. It's also about sharing. Sometimes it's about you've got a skill and you you've got one skill and someone else has got another skill. And it's about a collaboration between two different skills. (HAR06)

The feeling that you were doing something that is noble, and it has a value to, a social value and impact. It's a very good motivation. (HAR07)

Problems facing communities motivated volunteers on heritage projects, which were seen as improving people's lives. There were political motivators and volunteers felt involved in the ‘decision making’ connected to their heritage.

I have no idea about, you know, some of the problems that different communities are facing and how actually a local authority heritage project could help improve people's lives. (HAR05)

Certainly, the history and the members bringing their own histories to the group themselves. (HAR08)

I think it's political not party political, but it's certainly political because it's about the exercise, the power. I'm very interested in the devolution of power from the top of the pyramid down to the bottom, if you like, grass roots, grass roots involvement of people in being able to make decisions about their area and their own lives. That's always been a very strong motivator. (HAR09)

#### 3.1.2 Facilitators

Volunteers often had local access to heritage sites, which were likely to be community driven. The volunteers had connections to people who could access resources and funding to restore HAR sites was deemed helpful.

And if it was in York, it was very convenient because it being for the community, you had to actually live nearby. (HAR02)

Money to restore the bits that aren't in good shape and all that kind of stuff. So, having a group of people who are quite well connected and connected into resources or connected into people who potentially can access resources is a useful thing. (HAR10)

Volunteers were able to participate in their own time with flexible timetables.

One of the main reasons why it suited me was because you do it in your own time. You go out and do your surveys when you want to. (HAR11)

#### 3.1.3 Barriers

Retaining volunteers was problematic, there were deficient resources regarding management, and bureaucracy. It was identified that *core staff* would be helpful to support volunteers. The location of the heritage project was influential as affluent areas appeared more likely to receive funding, highlighting the lack of support for underfunded areas.

And we did get people beginning, but over time, we lost them. (HAR12)

I do feel that because, you know, I know it's being ran as a charitable trust organisation. But still, you need some core staff. (HAR13)

If the building were in Islington, in north London, for example, we wouldn't be doing this. It would never have been under threat. And it would have lots and lots of local money being thrown at it. Because we're in a poor, largely immigrant area of Birmingham. We've had to fight to draw attention to the importance of the building. (HAR14)

Threats to restoring heritage sites was finding money for restoration and the stress of dealing with bureaucracy. There were problems of managing grants and frustration at the pace of work.

You know, one of the big threats through it is money and it's finding money and money to look after it. Money to restore the bits that aren't in good shape and all that kind of stuff. (HAR10)

Negative impacts, as I I've said things like the stress of dealing with the bureaucracy, the accountants and all that that, that, they're negative impacts. (HAR15)

I feel a little bit frustrated at the pace of things. (HAR11)

Transport and community infrastructures negatively affected volunteers reaching heritage sites.

Infrastructure like public transport. It has a knock‐on effect to being able to visit the sites. (HAR16)

Younger volunteers were in the minority, perhaps because they and others were too busy managing family, work, or social commitments. Volunteers could only clear sites to make access better in certain months of the year.

I think there's a difference in age for somethings. (HAR08)

I enjoy doing it. But I'm pretty involved with the community as it is. We've got eight grandchildren, a big family. So, I'm not sure that I'm particularly keen to take on further responsibilities. (HAR17)

We can't do so much between, say, March and September because of the bird nesting. (HAR18)

Physical health problems, or domestic and financial responsibilities created barriers for volunteering. Not all volunteers, especially the younger ones, could afford to give their time unfunded and acknowledged some staff were paid.

No. No, well, as I said, if I could do more, I would do more. But I can't use my hands, my hands. They're not good. I can't bend. It's not only old age. As I said it's just health reasons, that's why I can't do anything. But I would love to get involved in it more. (HAR19)

She left…some of the younger people need to be paid because obviously, you know, I'm in a fortunate position that I can afford some of my time for free. (HAR20)

Volunteers expressed they felt threatened for their safety out at night in the fields if farmers were shooting and organising events in bad weather, in slippery farm tracks. Due to COVID‐19 there were many restrictions, causing disruption to projects.

We don't want to be out there when they're firing at night. (HAR21)

It is unfortunately, sadly, due to Covid‐19. Everything was disrupted to a great extent. (HAR05)

### 3.2 Theme 2—Being: Identity and belonging as a volunteer

Volunteers were fond of their surroundings, and they enjoyed their friendly, rural peaceful locations, diversity, and local history. Volunteers said they did not enjoy living in isolated industrialised and polluted areas.

I like the rural setting. And it is quiet. It's peaceful. It's friendly. (HAR21)

So, what don't I like about it? Just down the road 10 miles away is Teesside, which is very industrialised and that's not always a pleasant place to drive through. It's just there's been quite a large steelwork that's been closed for about five or six years. That's in a ruinous state. It just looks a mess. (HAR11)

It was cathartic cutting bracken and volunteering was rewarding and made some feel connected; it was important to preserve history for future generations and pass on crafts employed in constructing heritage buildings.

So, we're going out and cutting bracken, that's very satisfying in a sort of mindless way, really, going out and swinging a jungle life of bracken (laughs). I think it's very cathartic. (HAR22)

Makes me feel quiet, connected. (HAR20)

I think it's important that we do our best to preserve that heritage as much as we can and for the future generations to be aware of what's happened over this area over the years. But for current generations as well, I think we can learn a lot from it, if only through some of the crafts that are employed in constructing buildings, constructing monuments. (HAR11)

### 3.3 Theme 3—Capacity: Learning and diversifying experience

Volunteers gained new skills and enjoyed learning about history and archaeology and the application of skills they already had.

I enjoyed getting the experience and learning how things are done on an archaeological dig. (HAR02)

I also got involved in the management of the donations and some of the spreadsheets and stuff. And again, that's some skill I already had that I was able to apply. (HAR23)

It wasn't an outdoors type of activity. It was a desk. Yeah, it was a desk and remote. (HAR05)

HAR projects gave their volunteers an opportunity to gain skills and increase social engagement in a group of people with different types of abilities and an opportunity for volunteers *to think creatively*.

So, sending information on. And that sort of side of things. Trying to think creatively. (HAR21)

Other participants did not feel they had *learnt* any new skills, it was about *bringing knowledge* to the volunteering role.

No, I think it was more about bringing knowledge than skills. I can't pinpoint anything that I've learnt really. (HAR22)

### 3.4 Theme 4—Sharing: Community engagement, connectedness, and inclusivity

Volunteers liked that their community had a strong sense of sentiment, cohesion, and identity.

It's [the community] got a very strong sense of cohesion and identity. So that's one thing I like. (HAR09)

What I like the most is that I live in a diverse community. (HAR20)

Volunteers also felt that the values were about local ownership and legacy from the heritage sites, in that the monuments would not be abandoned or ignored as they are important markers to the community. Volunteers were aware that their participation would develop or expand tourism.

So, I think really the values primarily are about, you know, about local ownership and respect, really. And hopefully that's something that's going to be the legacy that monuments aren't abandoned or ignored. But are felt important as, historic place markers for the community, I think. (HAR08)

We were featured last year in one of the national newspapers because it was seen as a good walk to go on. And it was mentioned, that. So, yes, I do think we do get walking tourists. (HAR24)

Volunteering and inclusivity were related to diversity and inclusion, specifically age, culture, ability, intersectional inclusivity, and gender. Inclusivity appeared crucial for diversity efforts to succeed and created an inclusive culture *addressing several grounds* of diversity (age, race disability) within HAR volunteers, addressing any inequalities.

I wouldn't normally meet such a range of ages and interests as well. (HAR04)

I think what I like about it. Woolwich is very multicultural area, and the Garrison Church is part of the institution, the army. (HAR25)

Yeah, and the fact that Tim wants me to do this, is good. That means, it covers several grounds my age, my race, the disability. (HAR25)

Volunteers highlighted the relevance of practicing policies of providing equal access to opportunities and resources for people, including other volunteers who might otherwise be excluded or marginalised, including physical or mental disabilities or belonging to minority ethnic groups.

Because I come from a different ethnic background, gender background, age background, I have a disability. And Daisy has got a disability as well? So, all these things they don't have. I bring to, to the table with me. (HAR25)

Through recruitment and spreading the word, volunteers would share their experiences of volunteering and the benefits recommending volunteering to their local communities and friends.

A lot of people can benefit from volunteering experience. (HAR07)

This also included promoting sites and heritage to the local community by advertising on local billboards, putting articles in the local newspaper, through social media and holding village events.

We have got a little website now and we have had a few people get in contact. We also have got a Facebook page and a Twitter account. And it's very interesting about the local cemetery. (HAR26)

Where there was lack of public awareness volunteers would talk to the public about their role in the project and explain *roughly* what they were doing and the expected outcomes for restoring the at‐risk project for the local community.

They usually see the badge and say, oh, you're a volunteer ranger. And some of them may not know what we do. And sometimes they'll ask. And then, you know, you can tell them. We tell them roughly what happens. (HAR02)

### 3.5 Theme 5—Self‐nurture: Physical, psychological, and social benefits

There were minor adverse effects on wellbeing such as getting thorns in their fingers, carrying chalk on the side of a hill, or working outdoors in all weathers, including excessive heat, rain, wind and freezing conditions.

Probably the least is getting thorns in your finger and isn't scratched to death. Both with the vegetation and the rain. So, we don't work in too bad conditions. (HAR04)

Carrying chalk on the side of a hill is a lot. (HAR27)

Some experienced the psychological stress of volunteering but felt satisfied *seeing things done*.

A little bit of stress. It's just more. A little bit of stress. Yeah, but then everything is stress. Yeah, I suppose the satisfaction of seeing things done. (HAR24)

Negative impacts on volunteering were also associated with not feeling part of the community.

I guess being negative, as I said before, it is not really being part of the community. (HAR28)

Volunteering promoted psychological and physical benefits and brought people together, alleviated loneliness, created an interest, lifted mood/depression and created a ‘buzz’.

The museum's done good. Because I got a bit of a buzz from that. (HAR15)

This depression, it lifts the mood. (HAR18)

I enjoy, I come away feeling, you know, I've done something worthwhile in the day. And we said, you know, we've seen people. (HAR18)

Volunteering promoted physical activity and it was an opportunity for volunteers to walk outdoors, get fresh air, swim at the restored local baths, or do some restorative physical work such as lifting wheelbarrows and cutting bracken.

I think it's become much more involved and personal because it takes effort to lift barrow loads of chalk up and down hillsides and stamp all down. And so, it's physical, some blood, sweat and tears. (HAR29)

Benefits to wellbeing outweigh disadvantages, and volunteers felt rewarded and appreciated, working relationships strengthened and there was a core group of volunteers. Volunteering increased social interaction and friendships including quantity of relationships.

Any perceived disadvantages are far outweighed by the advantages. (HAR23)

And it's rewarding basically because people enjoy and appreciate your involvement. It's nice to feel appreciated. (HAR08)

There's a core group of people who are there most times. (HAR04)

### 3.6 Theme 6—Self actualisation: Retrospect and prospect

On personal reflection some felt they had not achieved anything personally as it is a team effort. For others what they learnt about the heritage sector potentially opened new possibilities for different types of HAR volunteering roles.

Sort of finding out and learning a bit more about the nuts and bolts of heritage itself and what it takes to look after a building. What are the, you know, I spend a lot of my time fundraising, but I didn't know the heritage sector as well as I know other sectors. So, you know, on many levels, it sort of opens new possibilities. (HAR10)

I don't think I've achieved a lot personally because it's too early. It's, it's a real team thing. (HAR15)

Restoring heritage sites made them more interesting to the community and changed local perceptions of the site. Volunteers would be happy to volunteer again in the future in the same project or other similar HAR sites and projects, as well as recommend volunteering to others.

So, it wasn't such an interesting site to look at from the local point of view. Quite the opposite. So, it has. I am sure, changed the local perception of the site. (HAR12)

It's been such a positive experience. It's kind of encouraged me to tell people about the project. (HAR28)

## 4 DISCUSSION

### 4.1 Summary of main findings

Volunteering was associated with positive physical, social and psychological wellbeing outcomes and confirmed the existing theory that Systematic Grounded Theory has enabled us to build theory on existing knowledge.^14^ Different types of wellbeing outcomes of the HAR volunteer experience were linked to an individual's motivation to volunteer and the characteristics of the site. Differing motivations were found at different ages, suggesting the importance of ensuring inclusion and diversity in HAR volunteers. Negative consequences on wellbeing of HAR volunteering were rarely observed in our findings.

Volunteers' sentiments about their roles, and the benefits gained differed between different HAR assets. For example, they ‘loved’ going outdoors to conserve heritage sites, while working on the reception desk at the local baths motivated volunteers to use the leisure facilities. There was a strong association with history, with ‘the members bringing their own histories to the group’, suggesting that HAR volunteering was associated with emotional wellbeing in ways which nonheritage volunteering was not. This helped to advance our understanding of the relationships between volunteering and wellbeing in relation to different HAR assets such as indoor museums, historical buildings, or landscape monuments.

This study demonstrated that HAR volunteering, community development and wellbeing were related as volunteers were able to identify ‘problems that different communities are facing’ and how a local heritage project could help ‘improve people's lives’. This suggested ways to embed and enhance wellbeing in communities through HAR projects. This showed that the volunteer's motivations were associated with needs of the local community and the heritage asset. It was also noted that all the types of HAR project sites were able to support volunteering and wellbeing.

### 4.2 Comparison with existing literature

The volunteer process model follows a series of stages exploring the volunteer experience.^18^ Stage 1 observes individuals' circumstances and motivation to volunteer. Stage 2 observes experiences and describes the relationship that develops between volunteers and organisations. Finally, Stage 3 explores consequences, such as a volunteer having positive or negative outcomes.^18^ Our findings broadly reflected this model. HAR volunteering was multifaceted in its wellbeing benefits at an individual level. For example, HAR volunteers' experienced improved mood associated with attachment to the heritage site, positive feelings about the environment, and widened social networks. Reflecting on their volunteering experience, HAR volunteers took pride in their work, felt they had achieved and experienced more than they expected, and felt valued as part of a team in the community. Many would recommend HAR volunteering to others.

The Taking Part Survey in 2018/2019 identified activities undertaken by volunteers in any heritage sector; 33.5% helped to organise or run an activity or event in any sector, 6.6% acted as trustees, 5.8% engaged with conservation or restoration and 1.8% of volunteers acted as stewards at a heritage site, museum or gallery.^4^

The condition or location of the HAR site showed an association with different types of motivations for volunteering including protecting, preserving, and restoring. HAR volunteering created a combination of intrinsic or extrinsic rewards.^19^ Extrinsic rewards included the potential for career development, to learn, gain qualifications and skills, expand knowledge, and create experiences. Younger HAR volunteers were more likely to be motivated for extrinsic purposes, for example to progress careers or fulfil work placements. Our findings are in line with research suggesting younger volunteers often engage in volunteering as an extension of their other roles.^20^, ^21^

Extrinsic motivations were less connected to the at‐risk status of the site, while wellbeing was often linked to skills being gained through restoration and preserving the HAR monument or building. This was more likely observed in younger age groups as they were likely to volunteer to support their education and had less disposable time. This demonstrated differing motivations for volunteering in different age groups.^19^

Our findings are also in line with existing studies showing that older volunteers were more likely to be intrinsically motivated as they had more time to give and therefore were more likely to experience positive emotions compared to younger volunteers.^19^, ^20^ Past research showed that volunteering does improve physical and psychological wellbeing of older people by helping them maintain self‐esteem, life satisfaction, access to support systems, and activity levels.^20^, ^22^, ^23^

Veal et al.^24^ reported that volunteering promotes wellbeing and may provide an added purpose to life after retirement. Educating older adults about the health benefits of volunteering may also facilitate participation in both research and volunteering.^22^ While growing evidence documents strong associations between volunteering and improved health and wellbeing outcomes, less is known about the health and wellbeing factors that lead to increased volunteering.^22^

HAR volunteering demonstrated many wellbeing associations including social determinants and subjective wellbeing. Wellbeing is complex, multifaceted and can be defined by two relevant aspects. First, social determinants of wellbeing are objective factors that contribute to a person's potential for wellbeing and ability to flourish, with application at individual (lifestyle factors), collective (how well a community is doing) and population levels (wider socioeconomic conditions). Second, subjective wellbeing is an individual's own cognitive and affective evaluation of their life. It includes personal health, emotional resilience, supportive social relationships and a feeling of relevance and social justice; at the highest level, this is about how well we feel we are doing in our own lives. Wellbeing in this context is personal and subjective, and universally relevant.^2^

Van Willigen^21^ examined the impact of volunteering on physical and psychological wellbeing among older people and contrasted these benefits with those experienced by younger volunteers. Findings showed that older volunteers experienced greater increases in life satisfaction over time because of their high rates of volunteering hours compared to younger adult volunteers. Older adults experienced greater positive changes in their perceived health than did younger adult volunteers.^21^

The motivational factors of hospital volunteers included serving the community, improved well‐being and health, recognition among friends and family, connections, avoiding loneliness, and a feeling of self‐worth.^20^ The study sample consisted of adults aged over 50. These types of factors may be considered useful targets for interventions and policies aiming to increase volunteering, including in older adults.^20^, ^25^

Our findings are also in line with Historic England's six‐strand framework linking wellbeing and heritage^4^ that identified activities to improve wellbeing with different routes (volunteering, visiting sites, sharing, therapy, belonging and experiencing) identified as increasing social engagement and self‐esteem.^4^ Historic England also highlighted that wellbeing is a policy issue, politically and conceptually linked with addressing health inequality and social cohesion as a long‐term government priority.

This supports observations based on Activity Theory^26^ that the more time committed, the greater the impact of volunteering on wellbeing. Not only the number but also the range of activity is correlated with time committed, with a broader range of activity correlated with greater benefits.^27^ This could explain why volunteering amongst older people may have an increasingly positive effect on psychological and physical wellbeing over time.

Outdoor HAR volunteering was beneficial to wellbeing, with HAR volunteers enjoying working in wide open spaces, walking in beautiful surroundings on sunny days, in memorial and church gardens, and the moors and coastal regions. Changes in several indicators of physical health, health behaviours, and psychosocial wellbeing suggest the benefits of increased volunteering.^25^

The study HAR volunteers of different ages demonstrated ‘prosocial behaviour’, regarded as a ‘social glue’, enabling cohesion among people of different ages or characteristics which benefited all members of the group.^28^ Post^29^ stated that volunteering, altruism, and prosocial behaviour are antecedents to positive wellbeing, happiness, personal health and public health. This also demonstrated positive wellbeing associations across all HAR volunteers because they shared the same interest in preserving and restoring HAR sites which would not be observed in other types of volunteering. Furthermore, positive emotions experienced from HAR volunteering and the at risk‐status of the project were an example of an intrinsic reward, relating to ‘giving back to community’ and ‘doing things for the people’. These motivations were demonstrated across all age where heritage volunteers wishing to learn new skills volunteers (most often younger volunteers) experienced the same positive emotions experienced by those not volunteering to learn new skills.

HAR volunteering projects were associated with many wellbeing outcomes including the ‘buzz’ of hedonic^30^ pleasure from satisfying their psychological needs, interests, enhancing the visitor experience wellbeing of visitors and values in restoring historic sites. This is in line with the functional approach^31^ which identifies the importance of matching anticipated psychological benefits with an individual's motivation to volunteer.

In line with self‐determination theory,^32^ HAR volunteering was motivated by wanting to satisfy basic psychological needs for autonomy, relatedness and competence. Autonomy refers to freely choosing the activity, while relatedness refers to the need for social cohesion, and competence refers to feeling effective in a task, and reportedly increased social wellbeing, alleviating loneliness, and elevating mood.^32^ It was socially rewarding creating social engagement which resulted in friendships.

For HAR volunteers there were local, social and family connections and a need to connect with their ‘natural environment’ which motivated them to take part in HAR projects. Some HAR volunteers wanted to offer their skills, and this was often the only type of volunteering that provided an opportunity to use, learn or share their specialist skills (e.g., archaeology). ‘Preserving’ an asset from harm led to emotional benefits from an increased attachment to place, feelings of security or comfort gained from a long‐term perspective and enhanced attachment to others and through exploring intimate personal stories.^4^

HAR volunteers were aware that their participation would develop or expand tourism. Restoring museums has the potential to enhance visitors' psychological wellbeing including the wellbeing of the individual, or group (e.g., volunteer) that are impacted by the outcome of the project.^33^ This can be achieved by individuals adhering to the design of ‘museum experiences’ that are attractive, comfortable, restorative, comprehensible, participative, innovative and sustainable.^33^

Before COVID‐19, heritage volunteering numbers had increased significantly over the past decade. The number of English Heritage volunteers increased remarkably from 650 in 2010/2011 to 3562 in 2018/2019 and in 2018, 49,000 people volunteered for Heritage Open Days.^10^ Furthermore a 2020 heritage volunteering survey showed that 58% of volunteers planned to immediately return to their heritage volunteering post when their role restarted following the COVID‐19 pandemic.^10^

Considering aspects of volunteering including engagement and support across pandemic conditions maybe relevant for future interventions.^20^

HAR volunteering was often facilitated by heritage sites being locally situated with flexible work scheduling. Barriers to volunteering and negative outcomes were also observed. Adverse outcomes associated with HAR volunteering were often minor, for example, getting thorns in fingers and stress related to the project (e.g., staff not showing for duties, volunteering in freezing weather conditions or labour‐intensive work).

### 4.3 Strengths and limitations

Employing Grounded Theory and an inductive approach has helped us to identify and gain a deeper understanding of the associations between HAR volunteering and wellbeing. Our findings coincided with existing literature, therefore, supporting Grounded Theory methodology is appropriate ‘when there is a need for new theoretical explanations built on previous knowledge to explain changes in the field’^34^ ^(p.70)^.

An important confounder in examining the relationship between volunteering and wellbeing is the bidirectional relationship between psychological wellbeing and volunteering.^35^ The personal wellbeing model shows that volunteer work requires an investment of personal resources to the extent that those volunteers who score higher on self‐reported measures of psychological wellbeing may be more likely to volunteer and may also see an increase in psychological wellbeing as an outcome of the volunteer experience.^36^

### 4.4 Implications for stakeholders, practice and future research

Our findings identifying different wellbeing outcomes are associated with different types of HAR sites, have the potential to contribute to the development of a tool to measure wellbeing outcomes in HAR volunteering. This study supported Historic England policies to promote wellbeing in heritage projects.^2^ HAR projects need to consider inclusivity across age, gender and ethnicity, for example in supporting younger volunteers to engage in and benefit from volunteering.^36^

The HAR projects covered a range of different heritage assets and volunteering activities from different geographical regions to explore the association of wellbeing with different project attributes. This study focused on the association between different types of HAR activities and wellbeing outcomes rather than just concentrating on perceived benefits as in previous studies.^21^

Establishing a method to capture wellbeing in volunteers may help identify demographic, cultural and geographical differences. The link between volunteering and wellbeing has predominately been measured using longitudinal data.^36^ Developing valid measurement tools to assess and monitor wellbeing outcomes in HAR volunteering interventions may help tailor these to the specific needs of volunteers, by informing volunteers whether the HAR intervention suits their motivation for volunteering and achieves their wellbeing outcomes.

Dekel et al.^37^ suggested that future research needs to include a range of measures that capture the motivational and behavioural factors in younger people, such as self‐reported measures of volunteer engagement, psychological wellbeing and perceived organisational support. Their findings suggested that organisations may need to provide greater resources to support volunteer work engagement and wellbeing in younger adults.^37^ These findings may be useful for not‐for‐profit organisations on advising how to develop better recruitment and volunteer retention strategies for younger or unrepresented adults.

HAR volunteering may also be an avenue for social prescribing^38^ as a means of improving people's wellbeing. Museums and art galleries are also considered as sites for public health interventions, whilst across the UK partnerships already exists between cultural heritage, and health care services.^39^ In line with social prescribing, museums have the potential to develop a similar scheme.^40^

## 5 CONCLUSION

We could not conclude that HAR project activity was the sole cause of increased wellbeing. However, in line with the volunteer model^18^ we have addressed questions of motivation, observed experiences and described relationships that develop between HAR volunteers and types of project sites. We also explored negative outcomes from HAR volunteering experiences associated with wellbeing.

Wellbeing outcomes alone may have the potential to increase volunteers' interest in HAR projects, even if they had limited interest in the heritage asset at the outset. We have identified psychological variables to develop an intervention tool to support wellbeing and HAR volunteering effectively. There is a need to ensure that volunteering is more inclusive.

## AUTHOR CONTRIBUTIONS

Carenza Lewis and Niro Siriwardena designed the study. Joseph Akanuwe and Claudia Sima collected the study data. Julie Pattinson, and D.L. conducted the qualitative analysis. Julie Pattinson, and Despina Laparidou wrote the first draft of the findings. Julie Pattinson drafted the rest of the manuscript. Carenza Lewis, Niro Siriwardena, Julie Pattinson, and Despina Laparidou edited the manuscript. All authors read and approved the final manuscript.

## CONFLICT OF INTEREST STATEMENT

The authors declare no conflict of interest.

## Data Availability

The data comes entirely from qualitative interviews. The current study did not generate any data sets for analysis.

## References

[1] Historic England . Heritage and Society. 2020. Accessed November 11, 2022. https://historicengland.org.uk/research/heritage-counts/heritage-and-society/

[2] Historic England . A strategy for heritage and wellbeing (p. 4). Accessed November 11, 2022. https://historicengland.org.uk/content/docs/about/strategy-wellbeing-heritage-2022-25/

[3] Power A , Smyth K . Heritage, health and place: the legacies of local community‐based heritage conservation on social wellbeing. Health Place. 2016;39:160‐167. 27126363 10.1016/j.healthplace.2016.04.005

[4] Department for Digital, Culture, Media & Sport . Taking Part Survey: England Adult Report 2018/19. 2018. Accessed November 11, 2022. https://assets.publishing.service.gov.uk/government/uploads/system/uploads/attachment_data/file/955293/Taking_Part_Survey_Adult_Report_2018_19_V2.pdf

[5] Reilly S , Nolan C , Monckton L . Wellbeing and the historic environment. 2018. Accessed December 12, 2022. https://historicengland.org.uk/images-books/publications/wellbeing-and-the-historic-environment/wellbeing-and-historic-environment/

[6] Sofaer J , Gallou E . Places of joy: the role of heritage during the COVID‐19 pandemic. February 2022. Accessed February 7, 2023. https://historicengland.org.uk/whats-new/research/back-issues/places-of-joy-the-role-of-heritage-during-the-covid-19-pandemic/

[7] Heritage Fund . Improving wellbeing . 2019. Accessed February 7, 2023. https://www.heritagefund.org.uk/publications/wellbeing-guidance

[8] Historic England . Wellbeing and historic environment: why bother? 2019. https://historicengland.org.uk/whats-new/research/back-issues/wellbeing-and-the-historic-environment/

[9] World Health Organization . *Basic Documents: Forty‐Ninth Edition (including amendments up to 31 May 2019)*. 2020. Accessed February 4, 2023. https://apps.who.int/gb/bd/pdf_files/BD_49th-en.pdf

[10] Marshall L , Bibby J , Abbs I . The Health Foundation. November 2022. Accessed January 2, 2023. https://www.health.org.uk/news-and-comment/blogs/emerging-evidence-on-covid-19s-impact-on-mental-health-and-health

[11] Rosemberg C , Naylor R , Chouguley U , Mantella L , Oakley K . *Assessment of the Social Impact of Volunteering in HLF‐Funded Projects: Yr 3*. Heritage Lottery Fund. 2010. Accessed January 2, 2023. https://www.heritagefund.org.uk/sites/default/files/media/research/social_impact_volunteering_2011.pdf

[12] Glaser B , Strauss A . The Discovery of Grounded Theory: Strategies for Qualitative Research. Routledge; 1999.

[13] Strauss A , Corbin JM . Basics of qualitative research: Grounded Theory procedures and techniques. Modern Lang J . 1993;77(2):325. 10.2307/328955

[14] Strauss A , Corbin J . Basics of Qualitative Research: Techniques and Procedures for Developing Grounded Theory. Sage Publications Inc.; 1998.

[15] Lewis C . Heritage at risk and wellbeing in volunteers. 2022. Accessed January 5, 2023. https://historicengland.org.uk/whats-new/research/back-issues/heritage-at-risk-volunteering-and-wellbeing/

[16] Reilly S , Nolan C , Monckton L . Wellbeing and the historic environment. *Historic England*. 2018. Accessed January 5, 2023. https://historicengland.org.uk/images-books/publications/wellbeing-and-the-historic-environment/wellbeing-and-historic-environment

[17] World Health Organization . WHO, definition of an older or elderly person. January 1, 2023. Accessed January 7, 2023. https://www.scribd.com/document/190077600/WHO-Definition-of-an-Older-or-Elderly-Person

[18] Omoto AM , Snyder M . The context and process of volunteerism. Am Behav Sci. 2016;45:846‐867.

[19] Legault L . Intrinsic and extrinsic motivation. In: Zeigler‐Hill V , Shackelford TK , eds. Personality and Individual Differences. Springer International Publishing; 2020:2416‐2419.

[20] Huang G , Wang JY , Chien CH , Clinciu DL . Psychosocial factors influencing volunteering motivation: a pilot study in a Taiwanese healthcare volunteer program. J Hum Behav Soc Environ. 2023;33:382‐392.

[21] Van Willigen M . Differential benefits of volunteering across the life course. J Gerontol B Psychol Sci Soc Sci. 2000;55(5):S308‐S318. 10985302 10.1093/geronb/55.5.s308

[22] Wilson J , Musick MA . Work and volunteering: the long arm of the job. Soc Forces. 1997;76:251‐272.

[23] Young FW , Glasgow N . Voluntary social participation and health. Res Aging. 1998;20:339‐362.

[24] Veal B , Sadeq NA , Atkinson TJ , Andel R , et al. Who volunteers? Results from an internet‐based cognitive monitoring study of community‐based older adults. Health Educ Behav. 2023;50(3):359‐368. 35703406 10.1177/10901981221101355

[25] Nakamura JS , Lee MT , Chen FS , et al. Identifying pathways to increased volunteering in older US adults. Sci Rep. 2022;12(1):12825. 35896597 10.1038/s41598-022-16912-x PMC9328015

[26] Gubrium JF . Toward a socio‐environmental theory of aging. Gerontologist. 1972;12:281‐284. 5071302 10.1093/geront/12.3_part_1.281

[27] Musick MA , Herzog AR , House JS . Volunteering and mortality among older adults: findings from a national sample. J Gerontol B Psychol Sci Soc Sci. 1999;54b:S173‐S180. 10.1093/geronb/54b.3.s173 10363048

[28] Baker S , Cantillon Z . Safeguarding Australia's community heritage sector: a consideration of the institutional wellbeing of volunteer‐managed galleries, libraries, archives, museums and historical societies. Aust Hist Stud. 2020;51(1):70‐87.

[29] Post SG . Altruism, happiness, and health: it's good to be good. Int J Behav Med. 2014;12:66‐75. 10.1207/s15327558ijbm1202_4 15901215

[30] Ryan RM , Deci EL . On happiness and human potentials: a review of research on hedonic and eudaimonic well‐being. Annu Rev Psychol. 2001;52:141‐166. 11148302 10.1146/annurev.psych.52.1.141

[31] Clary EG , Snyder M , Ridge RD , et al. Understanding and assessing the motivations of volunteers: a functional approach. J Pers Soc Psychol. 1998;74:1516‐1530. 9654757 10.1037//0022-3514.74.6.1516

[32] Deci EL , Ryan RM . Self‐determination theory: when mind mediates behavior. J Mind Behav. 1980;1:33‐43.

[33] Šveb Dragija M , Jelinčić DA . Can museums help visitors thrive? Review of studies on psychological wellbeing in museums. Behav Sci. 2022;12(11):458. 36421754 10.3390/bs12110458 PMC9687250

[34] Grbich C . Qualitative Data Analysis. Sage; 2007:70.

[35] Thoits PA , Hewitt LN . Volunteer work and well‐being. J Health Soc Behav. 2001;42:115‐131. 11467248

[36] Haski ‐ Leventhal D , Bargal D . The volunteer stages and transitions model: organizational socialization of volunteers. Hum Relat. 2008;61:67‐102.

[37] Dekel G , Geldenhuys M , Harris J . Exploring the value of organizational support, engagement, and psychological wellbeing in the volunteer context. Front Psychol. 2022;13:15572. 10.3389/fpsyg.2022.915572 PMC9496652 36160559

[38] Historic England . Heritage and social prescribing. 2021. Accessed January 10, 2023. https://historicengland.org.uk/whats-new/research/back-issues/heritage-and-social-prescribing/

[39] Camic PM , Chatterjee HJ . Museums and art galleries as partners for public health interventions. Perspect Public Health. 2013;133:66‐71. 23308010 10.1177/1757913912468523

[40] Stickley T , Hui A . Social prescribing through arts on prescription in a UK city: referrers' perspectives (part 2). Public Health. 2012;126:580‐586. 22578297 10.1016/j.puhe.2012.04.001

